# The Influence of Pain Severity and Interference on Satisfaction with Pain Management among Middle-Aged and Older Adults

**DOI:** 10.1155/2016/9561024

**Published:** 2016-12-22

**Authors:** Tamara A. Baker, Jessica L. Krok-Schoen, Melissa L. O'Connor, Amber K. Brooks

**Affiliations:** ^1^Department of Psychology, University of Kansas, Lawrence, KS, USA; ^2^Comprehensive Cancer Center, The Ohio State University, Columbus, OH, USA; ^3^Department of Human Development and Family Science, North Dakota State University, Fargo, ND, USA; ^4^Wake Forest School of Medicine, Winston-Salem, NC, USA

## Abstract

*Background*. Health outcomes are often contingent on how effective the individual is able to manage existent illness-related symptoms. This is all the more relevant among chronic pain patients.* Objective*. This study aimed to identify indicators of pain treatment satisfaction among middle-aged and older adults (*N* = 150) receiving outpatient treatment from a comprehensive cancer center.* Methods*. Patients were surveyed on questions assessing pain treatment satisfaction, pain severity, and additional social characteristics.* Results*. Descriptive data showed that middle-aged adults reported more pain locations, greater pain severity, and less satisfaction with pain treatment. A multivariate model was specified, showing older adults being more satisfied with their pain treatment. For the middle-aged adults, treatment satisfaction was generally lower with greater pain severity. This counters that for the older adults, where treatment satisfaction remained consistent despite increased levels of pain severity.* Conclusion*. These findings address an important issue regarding how pain is experienced across the life course. This suggests that general assumptions cannot be made about the health outcomes of older adults. Beyond the descriptive definitions of pain, there remains the need to develop models that account for determinants that may account for the pain experience among a diverse adult population.

## 1. Introduction

The American Cancer Society suggests that more than 1.5 million Americans will be diagnosed with cancer in 2016 [1]. This statistic is particularly relevant when addressing the incidence of cancer among middle-aged and older adults. Reports from the Surveillance, Epidemiology, and End Results Program (SEER) show that for all new cancer diagnoses an estimated 26% are among those 65 to 74 years of age, compared with 24% and 14% among those 55 to 64 and 45 to 54 years of age, respectively [2]. Considering these statistics, there is the need to ensure that patients are provided with quality and equitable intervention and prevention options, while addressing how satisfied they are with their prescribed treatment regimen.

Patient satisfaction is a core dimension of healthcare quality and patient-centered care. Measures of satisfaction are prescribed at examining the extent to which patients' healthcare experiences complement their expectations of healthcare delivery [3]. The domains of patient satisfaction have received much attention in the treatment of patients, particularly those with a cancer diagnosis. In 2005, patient satisfaction was considered one of the National Cancer Institute's (NCI) sponsored Patient Navigation Research Program's four core outcomes to reduce disparities in cancer care. The details of cancer care including coordination of services, receiving treatment from multiple specialists, and management of coexistent behavioral and physical health symptoms make the delivery of cancer treatment an arduous task, particularly for those who simultaneously experience pain-related side effects. This may ultimately impact their quality of life, while diminishing their collective functional capacities [4].

Despite significant advancements in pain treatment therapies, nearly one in two cancer patients report their pain being undertreated [5]. With these outcomes, existent data are beginning to confirm the influence social and behavioral determinants have on how satisfied (or not) patients are with their pain treatment [6]. For example, patients who describe a positive communicative relationship with their healthcare provider [6, 7] are more likely to follow treatment recommendations, report greater pain relief, and experience less pain-related anxiety [7]. This suggests that satisfaction with pain treatment may depend less on symptom relief, but more on social and provider level factors that impact the degree of satisfaction [8, 9]. This is particularly relevant for older adults who are more likely to have their pain underdiagnosed and undertreated [10].

While the relationship between cancer pain treatment and patient satisfaction has gained considerable attention, there remains a paucity of empirical evidence documenting the influence a patient's age has on satisfaction with pain treatment. To broaden our understanding of pain treatment satisfaction, this study aimed to understand the influence that age (middle-aged versus older adult) and additional social determinants have on pain outcomes. This is significant considering that most data focusing on older adults assume a one-size-fits all approach. Determining the influence of age on satisfaction with pain treatment contributes to our understanding as to how this demographic characteristic is assimilated in treatment options, resources, and health outcomes.

## 2. Materials and Methods

### 2.1. Participants

Analyses were conducted from a multiyear project designed to determine existing social and behavioral constructs that influence the experience of cancer-related pain (due to diagnosis and/or treatment) in older non-Hispanic Black and White patients receiving services from an NCI-designated comprehensive cancer center in the southeast region of the United States.

Patients who self-identified as non-Hispanic Black or non-Hispanic White, were ≥55 years of age, rated their pain severity (cancer-related) as ≥4 (as scored by the Brief Pain Inventory (BPI)), were able to read and understand English, and were able to provide consent were included for study participation. Data were collected through patient interviews on measures assessing demographic, physical and behavioral health, and social indicators. All patients were approached (and recruited) by a research assistant (RA) during the patient's medical visit (in the waiting area) to determine their interest and eligibility for study participation.

Each interview lasted approximately 45 minutes and was conducted in a private area in the clinic. Respondents were compensated for their participation in the study. This investigation was approved by the cancer center's Protocol Review Monitoring Committee and the university's Institutional Review Board [11].

### 2.2. Measures

#### 2.2.1. Primary Outcome

*Satisfaction with Pain Treatment.* The American Pain Society's Patient Outcome Questionnaire (APS-POQ) is a 16-item measure used to quantify each patient's satisfaction with pain treatment. The APS-POQ has a total of four subscales: pain intensity, pain interference, satisfaction with pain management, and beliefs about pain and pain management. For purposes of this investigation, satisfaction with pain management subscale was examined as the primary outcome variable. Each question was referenced to the patient's satisfaction with their treatment of cancer-related pain. Questions were measured on a six-point numeric Likert scale, with higher scores indicating greater satisfaction with pain treatment. Response choices included the following: 0 = very dissatisfied, 1 = dissatisfied, 2 = slightly dissatisfied, 3 = slightly satisfied, 4 = satisfied, and 5 = very satisfied [12]. Descriptive data of patient satisfaction with pain treatment were similarly analyzed. The satisfaction with pain treatment assessment for this sample was found to have moderate internal consistency (*α* = 0.70).

*Health Variables.* A checklist of physical comorbidities assessed the presence of common medical illnesses (e.g., arthritis and diabetes mellitus). The pain interference (impact on daily functioning) subscale of the Brief Pain Inventory (BPI), a 32-item quantitative measure designed to assess clinical pain, was included in the analyses to determine how much pain interfered with daily activities (7 items; *α* = 0.90). Response items were measured on an 11-point Likert scale, with higher summed scores indicating more interference with daily activities. For purposes of this study, a mean (total) pain severity score (composite of four single pain items: current, average, worst, and least pain) was included in subsequent analyses. Response choices were rated on an 11-point numeric summated rating scale (0–10; high scores indicating greater pain severity) [13].

*Chronic Pain Self-Efficacy*. Self-efficacy to cope with chronic pain was measured with the 13-item Chronic Pain Self-Efficacy Scale (CPSS). This measure consists of two subscales: pain self-efficacy (PSE) and self-efficacy for coping with other symptoms (CSE). For this investigation, the two subscales were included as separate variables. The PSE subscale was found to be reliable (five items; range = 10–100; *α* = 0.70). Similarly, the CSE was reliable, with nine items (*α* = 0.84) assessing the ability to cope with other symptoms. Items for both subscales were summed into a composite score. Each question was scored on a 10-point numeric scale, with high scores denoting greater self-efficacy [14]. 

*Demographic Characteristics.* Five demographic variables were included in the analyses: age, race, sex, education, and marital status. Age was dichotomized at the median of 64.5. The resulting two age groups were labeled “middle-aged” (*n* = 79, age range: 55–65 years) and “older” (*n* = 71, age range: ≥65 years). Education was assessed as the total number of years of formal schooling. Marital status was assessed as married, living as married, separated, divorced, single/never married, or widowed. Race was examined via nominal categories, with those who identified as non-Hispanic Black/African American or non-Hispanic White/Caucasian included in subsequent analyses [11].

### 2.3. Statistical Analyses

For each measure, descriptive statistics were calculated for the total sample and by age group. Differences between age groups were evaluated using multivariate analysis of variance (MANOVA) for the continuous variables and chi-square tests for race and gender. Correlations between the health indicators (total pain locations, comorbidities, pain interference, and pain severity) and the self-efficacy indicators (self-efficacy for pain management and self-efficacy for coping with other symptoms) were calculated to check for multicollinearity (results are not presented). A hierarchical multiple regression model was used to examine age group, race, sex, years of education, total pain locations, comorbidities, pain interference, pain severity, self-efficacy for pain management, and self-efficacy for coping with other symptoms as independent predictors of patient satisfaction with pain management.

To explore potential moderating effects of age group, interactions between age groups and the health (total pain locations, comorbidities, pain interference, and pain severity) and self-efficacy (management of pain, coping with other symptoms) indicators were also included in the model. Demographics were entered in Step 1, health and self-efficacy variables in Step 2, and interactions in Step 3. All continuous variables were converted to *z*-scores, thus allowing the variables to be centered for forming interaction terms and be equivalently scaled. None of the variance inflation factor (VIF) values for the predictors or interactions exceeded five, which is commonly used as a cutoff for high multicollinearity [15].

## 3. Results

### 3.1. Demographic Characteristics

The sample included older non-Hispanic Black and non-Hispanic White patients (*N* = 150), with a mean age of 65.4 ± 7.7 years. More than half the total sample was female (57%) and self-identified as non-Hispanic White (82%). Patients reported living with an average of two chronic medical conditions (2.7 ± 2.2) in addition to cancer. For the total sample, patients reported moderate levels of pain severity (4.01 ± 1.92; 0–10), with comparable reports for pain interference (33.2 ± 17.2; 0–10). Similarly, participants rated to be relatively satisfied with their pain treatment (13.1 ± 2.21; 0–5).

When the sample was dichotomized by age group, older adults reported a slightly higher level of educational attainment than that of middle-aged cohort (14.1 ± 2.42 versus 13.0 ± 2.41). Results from the descriptive analyses showed that the middle-aged adults reported more locations (2.57 ± 2.95 versus 1.94 ± 1.38) and pain severity (4.14 ± 1.82 versus 3.87 ± 2.03) than the older adults. Compared to the middle-aged adults, older adults reported to be more satisfied with their pain treatment (13.55 ± 1.79 and 12.69 ± 2.50). Other demographic and health characteristics, for the total sample and by age group, are provided in Table 1.

### 3.2. Multivariate Model

Results from the hierarchical regression model showed that each step was statistically significant at Step 1 (*R*^2^ = 0.09, *p* = 0.03), Step 2 (*R*^2^ = 0.16, *p* = 0.03), and Step 3 (*R*^2^ = 0.23, *p* = 0.03). Significant predictors included age group (B = 1.16; SE = 0.43; *p* < 0.01), pain severity (B = −1.07; SE = 0.40; *p* < 0.01), and the interaction between age group and pain severity (B = 1.31; SE = 0.57; *p* = 0.02) (Table 2). Data showed that older adults reported greater patient satisfaction with pain management than middle-aged adults, and pain severity was inversely associated with patient satisfaction with pain management.

**Figure 1:**
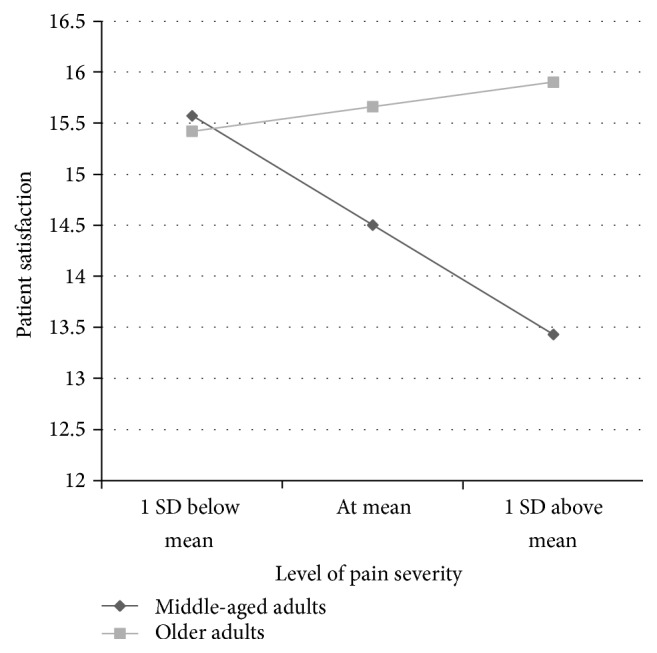
Estimated means of patient satisfaction with pain management by age group at different levels of pain severity.

This interaction is represented in Figure 1, in which estimated means of patient satisfaction with pain management are plotted by age group at three levels of pain severity (one standard deviation below the mean, at the mean, and one standard deviation above the mean) (Figure 1). Results show that for middle-aged adults patients' satisfaction with their management of pain was generally lower when pain severity was higher. However, for older adults, patient satisfaction with pain management remained consistent regardless of pain severity. When age was entered into the model as a continuous variable instead of being dichotomized, the same pattern of results emerged.

## 4. Discussion

This study aimed to explore how demographic, health, and other social indicators may differentially relate to satisfaction with pain management among a sample of middle-aged and older adults diagnosed with cancer. Findings from this study show the complexity of the pain experience, along with the importance of accounting for a range of factors that may influence how adequately pain is reported and recorded, the response to pain severity, and how satisfied patients are with their prescribed treatment of pain.

Results from this investigation showed that patient satisfaction with pain management was high for the total sample. This corroborates with previous studies showing that patients' satisfaction with pain management is consistently skewed in a positive manner even among those with high pain severity [16–20]. Beck et al. [17], for example, explored the paradox of high levels of satisfaction and increased levels of cancer-related pain. Patients identified four themes regarding the quality of pain management: being treated right, having a safety net, being in a partnership with their healthcare team, and having pain treatment that was efficacious. The added benefit of these data (themes) underscores the importance of acknowledging and understanding what contributes to a patient's source of satisfaction. Clarifying what factors reflect a patient's opinion of how satisfied she/he is with their pain treatment allows researchers and clinicians to identify the areas of improvement around cancer pain management and, subsequently, patient satisfaction.

High satisfaction with pain management, despite the presence of cancer-related pain, may also be related to the responsiveness and pain symptom monitoring of the cancer center from which the patients are receiving treatment. However, one must caution about drawing erroneous conclusions that problems, with pain management, stem specifically within the institutional setting. As noted, each system has a prescribed infrastructure for treating patients, which is based on a number of factors. Yet, for any treatment facility, the ultimate goal is that patients are treated equitably and are provided the necessary care to remedy the resultant pain.

Even with this explanation, another possible reasoning for this investigation's findings of high satisfaction reflects that of the American Pain Society Committee's (1995) report [12], suggesting that while individuals may report high levels of pain severity, the extent to which the painful experience interferes with various activities may not be a clinical concern to patients, thus accounting for high satisfaction despite the pain ratings. This is supported by the moderate pain interference with daily activities variable among this investigation's total sample, where pain interference did not emerge as a significant indicator of satisfaction with pain management. This outcome is all the more important, particularly when addressing the needs of the middle-aged and older adult populations. Some of these individuals may be dealing with more exigent issues, such as familial obligations (e.g., working and taking care of an elderly parent); that does not allow pain (despite the severity) to restrict their day-to-day obligations.

### 4.1. Age Group Differences in Patient Satisfaction

In addition to findings from the total sample, further interesting results showed that as pain severity increased, middle-aged adults reported significantly lower satisfaction with their pain treatment than their older counterparts. This result counters that of previous research showing no significant age-related association with overall satisfaction [18]. Other studies, however, have shown that an individual's age does have a significant influence on pain severity, response to pain, and adherence to symptomatic outcomes and treatment [21–23]. When assessing these age-related differences, attitudes of aging should also be considered.

It is similarly reported that those experiencing increased cancer-related pain are at a higher risk of functional capacities and psychological distress [24, 25]. Therefore, the reported age differences in patient satisfaction with pain management may be due to other (unmeasured) factors such as psychological distress, which is known to be associated with cancer pain [24, 25]. There is a growing body of literature documenting age group differences in reports of cancer-related pain and psychological distress [21–23, 26–28]. Wenzel et al. [28] found that older adults are better able to adjust psychologically to chronic illnesses than are their younger counterparts. Data similarly confirms that younger patients often report lower emotional acceptance of a diagnosis, poorer quality of life, and more psychological distress than older patients [27, 28]. These study findings are supported by the Socioemotional Selectivity theory which posits that over time older adults are more resilient and less reactive to stress (stressful situations) [29]. The presence of this age-related emotional regulatory response may provide the older adult with more effective methods of coping with cancer-related pain.

This assessment of pain among middle-aged and older adults raises an important question: what does it mean to age? It is well recognized that society has certain (mis)perceptions of what is involved with the aging process. These commonly held opinions may dictate the older adult's own views of what it means to age, although an inevitable process [30]. Finding age-related differences (between middle-aged and older adults) showed the complexity and difficulty in explaining age-related factors as dynamic constructs within the context of health (pain). In addressing this factor, we need to question if experiencing pain is a “normal” part of aging. This raises another important and more immediate question that begins to mitigate the perceptions of what it means not only to age, but to age successfully with a painful chronic medical illness.

Although this study showed interesting findings, there are a few study limitations that must be acknowledged. First, this is a cross-sectional study; therefore changes over time or established causal relationships in reported pain severity and patient satisfaction with pain management could not be determined. Although patients were asked to report on their cancer-related pain, it may have been difficult for some to distinguish the etiology of their physical pain. Therefore, due to the cross-sectional methodology of the current investigation, it was difficult to determine if the patient's pain was due to the cancer diagnosis. It is important to address this issue, particularly when examining the pain experience among an older adult population, as they are more likely to be diagnosed with multiple painful morbidities. Another limitation is that the majority of the total sample was White and well-educated, thereby limiting generalizability of some of the study's findings. However, the sample did include individuals with various cancer diagnoses, prognoses, and treatment regimens, which may increase generalizability to patient populations receiving care at a comprehensive cancer center. Finally, the data was collected via self-reports and may result in potential reporting bias (e.g., social desirability). Reactions to social desirability may have yielded responses favorable to the patient (lower pain severity) or the cancer center (higher satisfaction).

## 5. Conclusion

This study aimed to explore how demographic, health, and identified social indicators may serve as a facilitator and/or barrier to the satisfaction with pain treatment among middle-aged and older adults diagnosed with cancer. There are several clinical implications resulting from this study. First, middle-aged and older adults' pain should be regularly measured, monitored, and addressed. One area that needs to be considered is the patient's expectations of their pain experience and relief [31]. Similarly, there is the continued need to adequately assess patient satisfaction in their judgement with quality of care. Jean-Pierre et al. [32] have made concerted efforts in designing an assessment tool that acknowledges patient satisfaction, particularly among diverse cultural and socioeconomic populations of cancer patients. This suggests an awareness of the needs of the patient, while necessitating consistent assessment from screening to diagnosis.

Yet, it is important that healthcare providers discuss and assess their patient's knowledge and concerns regarding treatment satisfaction to determine the influence expectations (about symptom relief) may have on his/her satisfaction with quality of care. In addition, empowering patients and healthcare professionals about pain management may enhance the patient's outcomes, such as self-efficacy and perceived control over their pain.

This study clearly demonstrates the importance of considering age differences in measuring patient satisfaction with pain management and treatment. Further research is necessary to determine what strategies are needed in reducing the experience of chronic pain, while providing resources that are beneficial to all. Yet, while providing more insight into the pain experience among the adult population, there remains the lack of detail as to* why* these similarities and/or differences occur.

One area that remains unexplored is identifying the influence social determinants of health (SDoH) have on the pain experience. SDoH take a dynamic approach to understanding the circumstances by which the patient deals with pain, as opposed to blaming the patient for not being able to successfully manage their pain. Addressing the SDoH domains may provide a more cohesive understanding to the experience and treatment of pain across economic, race, age, and gendered groups.

## Figures and Tables

**Table 1:** Descriptive statistics for total sample and middle-aged and older adults.

Variables	Total (*N* = 150)		Middle-aged adults (*n* = 79)		Older adults (*n* = 71)	
	M ± SD	%	M ± SD	%	M ± SD	%
Age^*∗*^	65.38 ± 7.72		59.58 ± 3.01		71.83 ± 6.05	

Race (% White)		82%		77%		87%

Gender (% female)		57%		58%		56%
Years of education	14.03 ± 2.41		13.91 ± 2.41		14.17 ± 2.42	
Total pain locations	2.23 ± 2.34		2.57 ± 2.95		1.94 ± 1.38	
Comorbidities	2.68 ± 2.21		2.51 ± 2.37		2.87 ± 2.01	
Pain interference	33.23 ± 17.25		35.74 ± 16.30		30.38 ± 17.97	
Pain severity	4.01 ± 1.92		4.14 ± 1.82		3.87 ± 2.03	
Self-efficacy for pain management	23.12 ± 9.47		22.47 ± 10.57		23.83 ± 8.13	
Self-efficacy for coping with other symptoms	39.85 ± 15.47		37.90 ± 16.72		41.90 ± 13.88	
Patient satisfaction with pain management^*∗∗*^	13.10 ± 2.21		12.69 ± 2.50		13.55 ± 1.73	

^*∗*^Significantly different between age groups at *p* < 0.05.

^*∗∗*^Significantly different between age groups at *p* < 0.01.

**Table 2:** Predictors of patient satisfaction with pain management (final model).

Variables	B	SE	*p*	VIF
Age group	1.16	0.43	<0.01^*∗*^	1.16
Race	0.37	0.65	0.51	1.15
Gender	0.04	0.45	0.93	1.23
Years of education	−0.36	0.23	0.13	1.13
Total pain locations	0.14	0.22	0.53	1.36
Comorbidities	0.27	0.27	0.33	1.88
Pain interference	0.38	0.39	0.33	3.50
Pain severity	−1.07	0.40	<0.01^*∗*^	3.22
Self-efficacy for pain management	−0.31	0.32	0.34	2.53
Self-efficacy for coping with other symptoms	0.61	0.36	0.09	3.00
Age group × pain locations	−0.30	0.61	0.62	1.39
Age group × comorbidities	0.42	0.45	0.35	1.81
Age group × pain interference	−0.41	0.54	0.46	3.49
Age group × pain severity	1.31	0.57	0.02^*∗*^	3.33
Age group × self-efficacy for pain management	0.52	0.51	0.31	2.29
Age group × self-efficacy for coping with other symptoms	−0.30	0.54	0.59	2.58

^*∗*^ *p* < 0.05.

*Note*. The reference group for age group was middle-aged adults, and the reference group for race was Whites. All continuous variables were *z*-scored. VIF = variance inflation factor.
